# Biological efficacy of simulated radiolabeled Lipiodol® ultra-fluid and microspheres for various beta emitters: study based on VX2 tumors

**DOI:** 10.1186/s13550-023-01051-9

**Published:** 2023-11-23

**Authors:** Arnaud Dieudonné, Stéphanie Becker, Miguel Soares, Claire Hollenbeck, Marie-Christine De Goltstein, Pierre Vera, Robin Santus

**Affiliations:** 1 Nuclear Medicine Department, Henri Becquerel Cancer Center, 76000 Rouen, France; 2 QuantIF-LITIS EA4108, University of Rouen, Rouen, France; 3 Research and Development Division, Laboratoire Guerbet, Aulnay-Sous-Bois, France

**Keywords:** VX2, Dosimetry, SIRT, Lipiodol, Microspheres

## Abstract

**Background:**

Radioembolization is one therapeutic option for the treatment of locally early-stage hepatocellular carcinoma. The aim of this study was to evaluate the distribution of Lipiodol® ultra-fluid and microspheres and to simulate their effectiveness with different beta emitters (^90^Y, ^188^Re, ^32^P, ^166^Ho, ^131^I, and ^177^Lu) on VX2 tumors implanted in the liver of 30 New Zealand rabbits.

**Results:**

Twenty-three out of 30 rabbits had exploitable data: 14 in the group that received Lipiodol® ultra-fluid (group L), 6 in the group that received microspheres (group M), and 3 in the control group (group C). The histologic analysis showed that the Lipiodol® ultra-fluid distributes homogeneously in the tumor up to 12 days after injection. The X-ray μCT images showed that Lipiodol® ultra-fluid has a more distal penetration in the tumor than microspheres. The entropy (disorder of the system) in the L group was significantly higher than in the M group (4.06 vs 2.67, *p* = 0.01). Equivalent uniform biological effective doses (EUBED) for a tumor-absorbed dose of 100 Gy were greater in the L group but without statistical significance except for ^177^Lu (*p* = 0.03). The radionuclides ranking by EUBED (from high to low) was ^90^Y, ^188^Re, ^32^P, ^166^Ho, ^131^I, and ^177^Lu.

**Conclusions:**

This study showed a higher ability of Lipiodol® ultra-fluid to penetrate the tumor that translated into a higher EUBED. This study confirms ^90^Y as a good candidate for radioembolization, although ^32^P, ^166^Ho, and ^188^Re can achieve similar results.

**Supplementary Information:**

The online version contains supplementary material available at 10.1186/s13550-023-01051-9.

## Background

Hepatocellular carcinoma (HCC) is the most common primary liver cancer that develops from liver cells. It is the seventh most common cause of cancer worldwide and the second most common cause of cancer death [1]. Many treatments are used (from surgery to palliative treatment), selected according to the BCLC (Barcelona Clinic Liver Cancer) recommendations. Radioembolization is one option for treatment that has recently been introduced in the BCLC classification for early-stage patients [2].

The products currently approved for clinical use are glass microspheres (TheraSphere®, Boston Scientific), resin microspheres (SIR-Sphere®, SIRTEX Medical), both labeled with yttrium-90 (^90^Y), and polyglycolic acid-co-dl-lactic acid) (PGLA) microspheres labeled with holmium-166 (^166^Ho) (QuiremSpheres™, Terumo). The physicochemical characteristics of these different microspheres vary very slightly in diameter, but their main difference is their range of activity per microsphere, which is summarized in Table 1.

**Table 1:** Characteristics of products based on radioactive microspheres available in Europe for the treatment of liver tumors as of the first quarter of 2023

	TheraSpheres®	SIR-Spheres®	QuiremSpheres®
Microsphere diameter	15–35 µm	20–60 µm	15–60 µm
Material	Glass	Resin	Poly(glycolic acid-co-dl-lactic acid)
Isotope	Yttrium-90	Yttrium-90	Holmium-166
Activity per microsphere	70–2500 Bq	50–150 Bq	450 Bq
Approved area	Asia, Australia, Canada, Europe, USA		Europe

The quantity of microspheres injected varies according to the activity to deliver and the calibration of the delivered vial. Indeed, TheraSphere® can be injected with varying activity per sphere, from a maximum of 2500 Bq per microsphere at the time of calibration, down to 70 Bq at the expiration date (15 days after calibration). For SIR-Spheres®, the activity per vial orderable is between 3 and 10 GBq with a fixed number of microspheres, which leads to an activity per sphere between 50 and 150 Bq. This results in different biodistributions, which were described in the liver parenchyma [3] and impacts the acceptable dose limit in the healthy liver [3–5]. The impact on the tumor is also suspected to result in a different dose–response profile according to the microsphere load as suggested by Romanò et al. [6].

An alternative to the use of microspheres is to inject radiolabeled Lipiodol® ultra-fluid. This method was originally developed with Iodine-131 (^131^I) [7, 8] and was commercially available in the 2000s as Lipiocis® (CIS BIO International, Gif-Sur-Yvette, France). Radiolabeling of Lipiodol® ultra-fluid was also proposed with a rhenium-containing ligand dissolved in Lipiodol® ultra-fluid, the complex SSS, which stands for “Super-Six sulfur”. A Lipiodol® labeled with rhenium-188 (^188^Re) has been proposed [9].

The benefit of Lipiodol® ultra-fluid is that its biodistribution in the hepatic tumor has been described for years in the context of trans-arterial embolization. Its penetration to the venous sinuses, its extravasation, and its intra-cellular internalization give it a very high tumor coverage [10, 11]. The accumulation of Lipiodol® ultra-fluid in the tumor results from the specific characteristics of the tumor microenvironment described by Folkman [12]. The lack of contractility of the neovessel, the very slow blood flow [13, 14], and the increase in vascular permeability lead to an accumulation in the extracellular space.

Several radionuclides have been selected as candidates for radioembolization with Lipiodol® ultra-fluid [15] and are summarized in Table 2. They are all beta emitters and have a relatively long half-life (> 10 h). The value Δ_β_/λ is the ratio of Δ_β_ the average energy released per β disintegration and λ the physical decay constant of the radionuclide, which stands as the total energy releasable for a source of 1 Bq. For a given radionuclide, the β radiation-absorbed dose D_β_ can be calculated according to this value, with the assumption of low penetrating particles and high retention over time:

1 $${D}_{\upbeta }=\frac{{\Delta }_{\upbeta }/\uplambda \times A}{m}$$

with *A* being the activity administered in the target and *m* the mass of the target. Depending on the radionuclide, the activity leading to a given absorbed dose can go from 1 to 20-fold (see Table 2). In addition, the continuous slowing-down approximation (CSDA), i.e., the range of the β particles goes from 0.4 to 4 mm. These differences can have an impact on therapeutic effect. Indeed, for a given average absorbed dose, differences in the micro-scale absorbed dose distribution can cause variations in the anti-tumor effect.

**Table 2:** Radionuclides characteristics used in SIRT, data taken from [16] and NIST ESTAR Program [17]

	Physical half-life (h)	*β* released energy per disintegration Δ and CSDA range in water in parenthesis	Δ_β_/*λ* [10^−9^ J∙Bq^−1^]
Phosphorus-32 (^32^P)	343	696 keV (2.8 mm)	198
Yttrium-90 (^90^Y)	64	933 keV (4 mm)	49.6
IODINE-131 (^131^I)	192	182 keV (0.4 mm)	29.0
Holmium-166 (^166^Ho)	27	665 keV (2.6 mm)	14.9
Lutetium-177 (^177^Lu)	160	451 keV (1.5 mm)	59.8
Rhenium-188 (^188^Re)	17	762 keV (3.1 mm)	10.8

We propose to study these effects according to the radioembolization agent type and radionuclide using dosimetry and radiobiological modeling, to consider micro-scale heterogeneities and dose-rate effects. To this aim, we compared the biodistribution of Lipiodol® ultra-fluid with those of microspheres comparable in the rabbit hepatocarcinoma model (VX2). For each explant, the dosimetry was modeled for the following radionuclides: ^32^P, ^90^Y, ^131^I, ^166^Ho, ^177^Lu, and ^188^Re.

## Materials and methods

### Animals

All animal experiments were conducted in compliance with European Union Directive 2010/63/EU on the protection of animals used for scientific purposes. The protocol was approved by the local animal research ethics committee. All surgeries were performed under general anesthesia and aseptic conditions and were supplemented by appropriate analgesic programs.

The VX2 rabbit tumor is a commonly used animal model for translational research on HCC in interventional radiology [18]. Implantation of a VX2 fragment was performed in healthy New Zealand white rabbits (Charles River Laboratories, Saint-Germain-Nuelles, France).

VX2 well-vascularized tumor fragments (25 mg) were sampled from a carrier animal and immediately implanted in the left median lobe of the exposed liver of the recipient rabbits. One donor was used for 3–6 receivers. Tumor growth lasted at least 19 days after implantation. Ultrasound imaging was performed to ensure that the tumor had reached a length of at least 10 mm (major axis); otherwise, the animal was kept until the tumor was workable. Nineteen to twenty days after tumor induction, the population was divided into 3 groups: L for Lipiodol®, M for microspheres, and C for control.

### Interventional procedure

The rabbits of the L and M groups received buprenorphine (Buprecare® 0.14 mL/kg) 1 h before surgery and were hydrated with 50 mL of saline subcutaneously in the flank. Then, they received an intravenous injection of heparin diluted to 1/10 at a dose of 50 IU/kg in the ear. A pediatric valve introducer 4F (Radifocus® TERUMO™) was inserted into the femoral vein and a 1.7F catheter (Microcatheter 1.7F angle 90° - ECHELON™ - MEDTRONIC EV3) was guided under x-ray angiography (Philips Veradius®) to the feeding artery of the tumor at the level of the left hepatic artery. After removal of the catheter, the skin and muscle planes were sutured at the paw level.

### Injection

The L group received an adjusted dose of Lipiodol® ultra-fluid into the left common hepatic artery up to reflux or pulmonary passage and to a maximum volume of 0.4 mL. The Lipiodol® ultra-fluid (Guerbet) injection liquid contains per 1 ampoule of 10 mL ethyl esters of iodized fatty acids of poppy seed oil, equivalent to 4.8 g of iodine (480 mgI/mL).

The M group received a fixed volume of 0.3 mL of microspheres in the same injection site. The radiopaque microspheres used in this study were made polyethylene glycol methacrylate (PEGMA) resin microspheres and were sieved to obtain an average diameter of 33 µm. They were made by Guerbet Research representative of approved microspheres in terms of size, which have been customized to make them radiopaque for the purpose of the study. Just before injection, 300 µL of microspheres were taken from the vial and suspended in 3 mL of saline water. The total amount of this suspension was injected slowly (about 0.1 mL∙min^−1^).

The C group received nothing.

### Imaging

Different time intervals were studied to investigate the distribution kinetics of the products. Because of its ability to extravasate leading to a possible modification of distribution during the first hours after injection, the pharmacokinetics of Lipiodol® ultra-fluid (L group) was studied at different timepoints (15 min (D0), 1, 2, 6, 9 and 12 days). For microspheres (M group) which are known to stay several months in the intravascular compartment, only the following delays were studied: 15 min (D0) and 12 days (D12) after injection. The C group was imaged at 15 min, 6 days, and 9 days. At studied time-points, the rabbits were euthanized by an intravenous injection of pentobarbital at a dose of 1 mL/kg under general anesthesia. The liver was explanted, and the tumor was isolated for high resolution 3D X-ray micro-computerized tomography (µCT). A Quantum GX2 (Perkin-Elmer) was used with the following parameters 90 kV, 88 µA, and a CuAl filter, and an acquisition time of 14 min. The field of view diameter was 72 mm or 86 mm depending on the size of the tumor, leading to a voxel side of 0.144 mm or 0.172 mm.

### Histology

For the L group, as soon as the µCT image was acquired, the tumor was cut into slices of up to one centimeter, frozen (− 80 °C) and sent for analysis to Oncovet Clinical Research (Clinical Research, Loos, France). Frozen samples of liver with tumor were cut into sections of 12 µm thick. The sections were stained with Hemalum-Eosin after a previous silver staining (2.5%, 60 min, 4 °C) allowing the detection of Lipiodol® ultra-fluid. Assessments from the resulting histologic slides were performed by a veterinary pathologist blinded to sample. The Lipiodol® ultra-fluid and microspheres distributions were studied in the vascular network and in the parenchyma of the tumors.

### Imaging analysis

To compare Lipiodol® ultra-fluid and microspheres capabilities to penetrate into tumor tissues, we applied a set of first-order radiomic features on the µCT images. To do so, the tumors were segmented manually using the software tool 3DSlicer [19]. The radiomics features were extracted using the SlicerRadiomics extension based on PyRadiomics [20]. A Spearman correlation test was done between time delay, tumor volume, and each radiomic feature. For these variables, the 3 groups were compared using the non-parametric Wilcoxon test. The statistical significance was considered to be achieved for a *p* value below 0.05.

### Dosimetry

Tri-dimensional (3D) dosimetry was modelized based on the Lipiodol® and microspheres distribution deduced from the µCT images. The tumor contours previously defined for the radiomic analysis were used. The distribution volume of iodine was segmented by manual thresholding. All voxels belonging to this structure were scaled so that the values were ranging from 0 to 1. The resulting image templates were then used to generate the activity maps so that the total activity within the tumors was equal to 1 MBq.

The activity in voxels was converted to time-integrated activity, which is also referred as the total number of disintegrations over the course of the treatment. In radioembolization, the calculation is simplified by the fact that the biological half-life is far greater than the physical half-life of the radionuclides used. Thus, time-integrated activity *Ã(s)* in each source voxels was calculated as

2 $$\widetilde{A}(s)=\frac{A(s,t=0)}{\lambda }$$

with *A*(*s*,*t* = 0) being the initial activity in the voxel and *λ* the decay constant of the radionuclide.

The absorbed dose was calculated in water using dose-point kernel (DPK) convolution implemented in a previous study [21, 22]. Water DPKs had a resolution of 0.1 mm. The dose *D(x)* at position *x* was calculated as

3 $$D\left(x\right)=\iiint \widetilde{A}\left(s\right){k}_{w}\left(\left|s-x\right|\right)\mathrm{d}s$$

with *s* being the position of the source, $\widetilde{A}\left(s\right)$ the time-integrated activity, and *k*_*w*_ the kernel in water.

The absorbed dose by tumor was calculated for each radionuclide in Gy per MBq administered to the tumor, which equals the ratio of *S* factor over the radionuclide decay constant *λ*. Indeed, according to the medical internal radiation dose (MIRD) formalism [23, p. 21], the tumor-absorbed dose is expressed as:

4 $$D=\widetilde{A}\times S$$

Hence, knowing that for radioembolization $\widetilde{A}=\frac{A}{\lambda }$, the tumor absorbed dose over the administered activity within the tumor can expressed as:

5 $$\frac{D}{A}=\frac{{S}}{\lambda }$$

To compare the biological efficacy between absorbed dose distributions, the biological effective dose (BED) was calculated according to the linear-quadratic model applied to radioembolization [24] as:

6 $$\mathrm{BED}=D\left(1+\frac{\lambda }{\lambda +\mu }\times \frac{1}{\alpha /\beta }\times D\right)$$

with μ the DNA repair constant, α and β are the linear and quadratic cell killing constants. We set the value of μ to 0.46 h^−1^ as reported by Cremonesi et al. for tumors [25], and α and β values to, respectively, 0.037 Gy^−1^ and 0.0028 Gy^−2^, as reported by van Leeuwen et al*.* [26].

To consider the heterogeneity of absorbed dose distribution, we implemented the equivalent uniform dose (EUD) concept of Jones and Hoban [27] to the BED leading to the EUBED:

7 $$\mathrm{EUBED}=-\frac{1}{\alpha }\mathrm{ln}\left({\sum }_{i=1}^{N}{e}^{-\alpha \times {\mathrm{BED}}_{i}}\times {v}_{i}\right)$$

with BED_*i*_ being the histogram *i*th bin, *v*_*i*_ the volume fraction, and *N* the number of histogram bins. EUBEDs were calculated for absorbed doses ranging from 1 to 1000.

### Statistics

Mean and standard deviation were calculated for the following variables for each radionuclide: tumor volume V, *S*/*λ*, EUBED(D = 100 Gy). The L and M groups were compared for each variable using the Kruskal–Wallis test by ranks. The statistical significance was set for a *p* value < 0.05. All statistics and graphics were processed using RStudio 2022.12.0.353 [28] and R 4.2.2 [29].

## Results

### Population

Thirty female New Zealand rabbits were included in the study (mean body weight 3.68 ± 0.35 kg), 19 in the Lipiodol® ultra-fluid group, 8 in the microspheres group, and 3 in the control group. Five animals were excluded from the L group due to: motion artifacts during imaging (1), sub-optimal image quality (1), tumor filling failure embolization, and highly necrotic tumor (3). Two animals were excluded from the M group due to a technical issue during injection.

### Histology

**Fig. 1:**
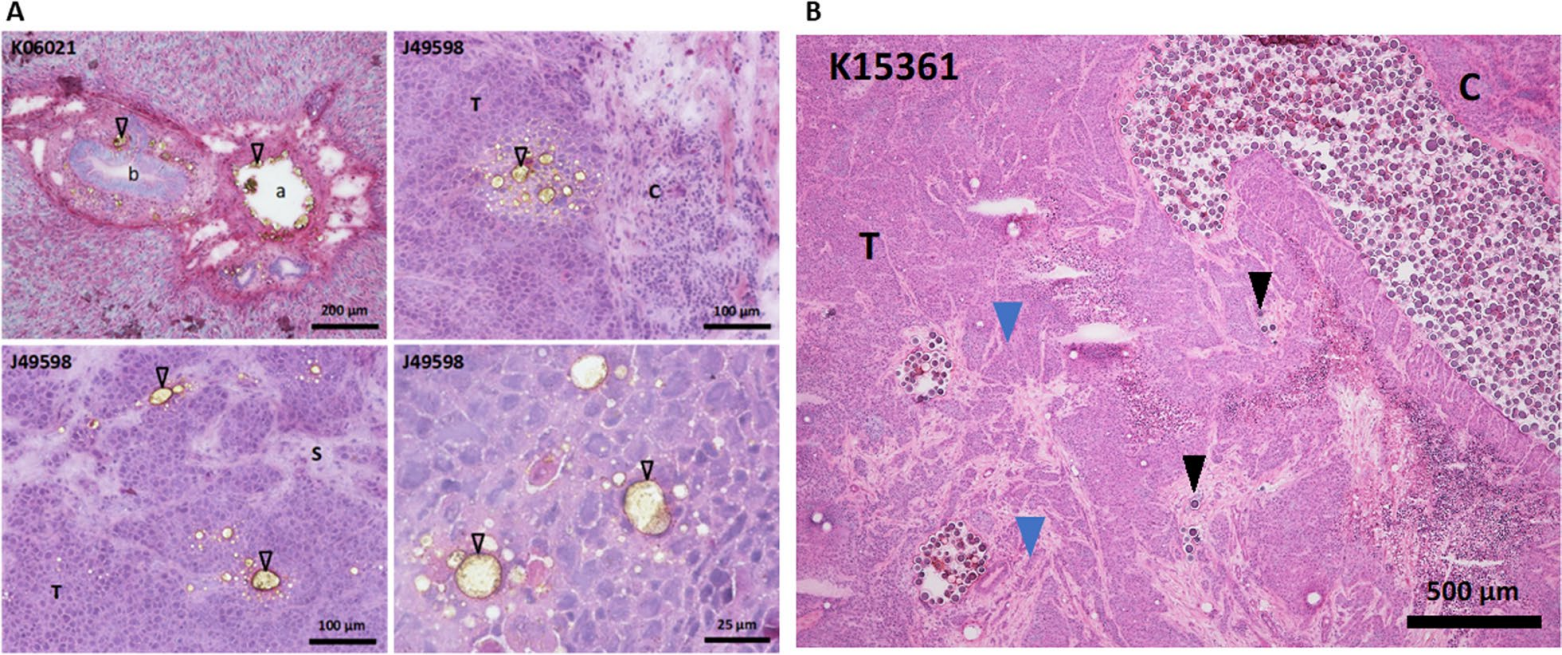
Histological analysis. **A** (group *L*): Stainable material was present in liver portal area, both inside main (a) and smaller portal vessels (open arrowhead). Biliary canal was indicated (b). Inside the tumor, some stainable material was present at the tumor periphery near the capsule (c) as clusters of irregularly sized stainable material-containing vacuoles and in the vicinity of larger tumor stromal trabeculae (s) identified due to their lower cellular content and lighter eosinophilic stain. **B** (group *M*): Cluster of microspheres filling the lumen of the main arteries (blue arrow) and isolated microspheres in small capillaries inside the tumor (black arrowhead)

The distribution kinetic of the L group shows that between 24 h and 12 days post-injection, the contrast remains homogeneously distributed in the hepatic portal zones, inside vascular structures of the tumoral capsule, and inside tumors, mainly in their peripheral stroma. Nevertheless, at 15 min post-injection, the distribution of Lipiodol® ultra-fluid appeared to be essentially intravascular for two rabbits (J49598/K23002) and partially intravascular for one rabbit (K06029), before the product extravasates at subsequent times. As expected, microspheres always remained purely intravascular at 15 min and 12 days. Histology images results are shown in Fig. 1.

### Imaging/radiomics

**Fig. 2:**
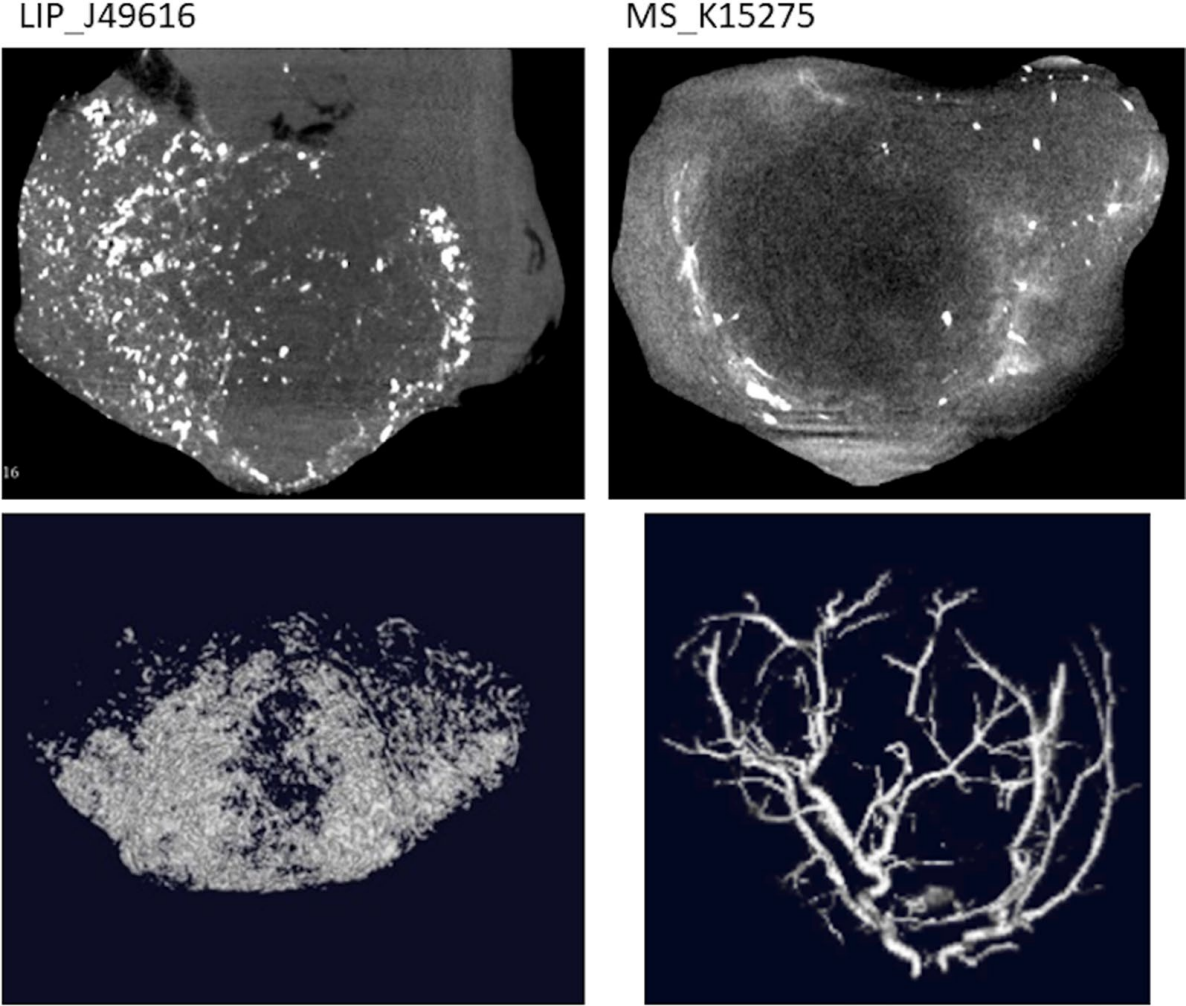
Transaxial slices (top) and 3D reconstruction of iodine signal (bottom) of tumors J49616 (left) that received Lipiodol® ultra-fluid (*L* group) and K15275 (right) that received microspheres (*M* group)

The analysis of the CT images allows for visual differentiation of the 2 groups: the L group presents higher density, not only in the large vessels, but also in the smaller structures and extravasated to fill the entire tumor parenchyma (except the central zone considered as necrotic), as can be seen in Fig. 2. In the M group, only large and mostly peripheral vessels showed contrast and penetration distal to the feeding vessels. This also translates into the entropy being significantly higher for the L group than for the M group (4.06 vs 2.67, *p* = 0.01)***.***

**Fig. 3:**
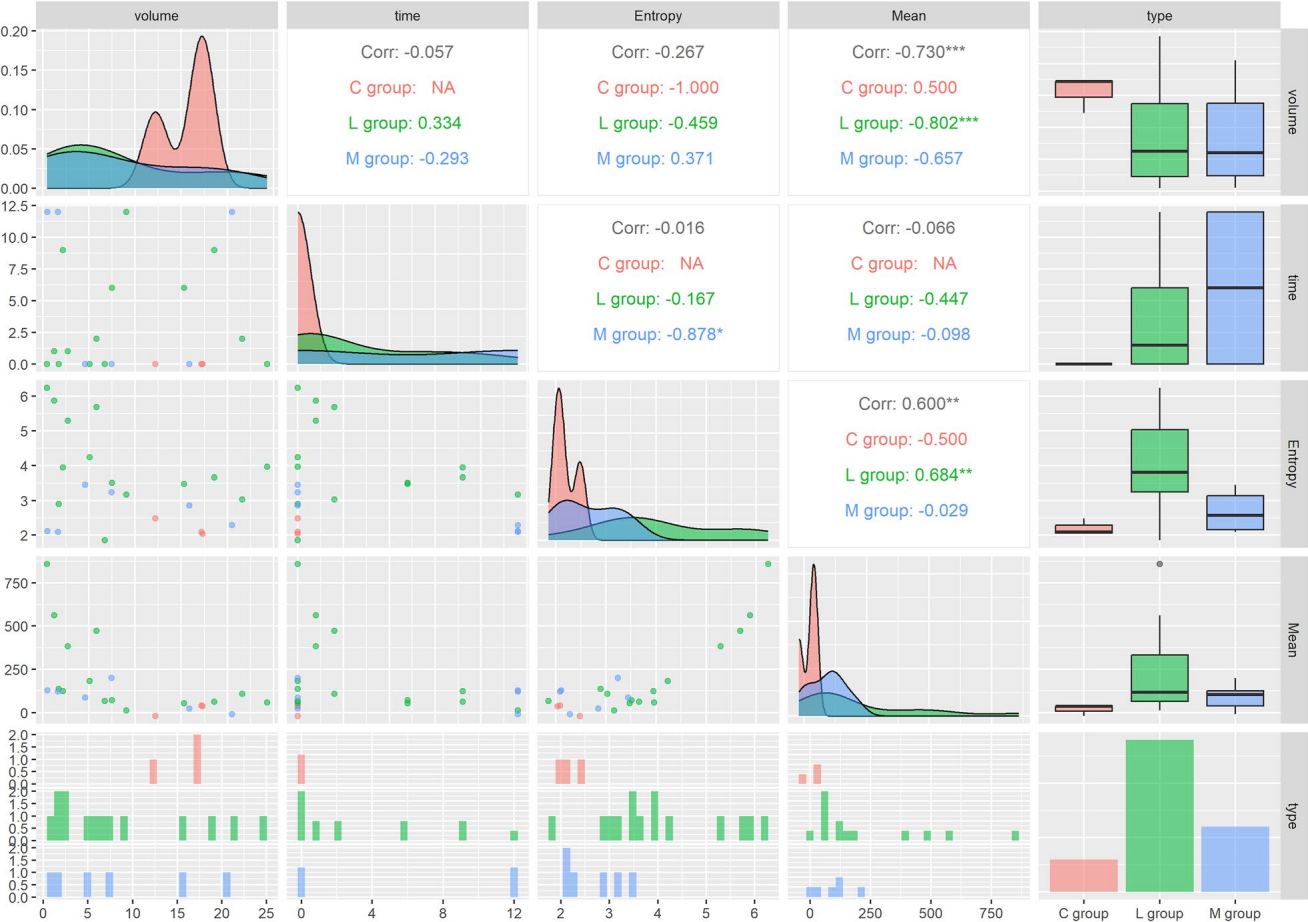
Density plots, scatter plots, boxplots, and correlation (spearman) for volume, time, entropy, and mean. The number of * symbols in superscript indicates the significance of the correlation value

Tumor volumes were 8.73 ± 8.0 mL for L group, 8.43 ± 8.1 for the M group, and 15.6 ± 2.9 mL for the C group (*p* = 0.26). The time delay between injection and imaging was 3.43 ± 4.1 days for L, 6.0 ± 6.6 for M, and 0.0 ± 0.6 for C (*p* = 0.64). A strong negative correlation was found between entropy and time for the M group (*r* = − 0.878, *p* < 0.05). The analysis of the radiomics results is presented in Fig. 3. Additional radiomic results are available as Additional file 1.

### Dosimetry

**Fig. 4:**
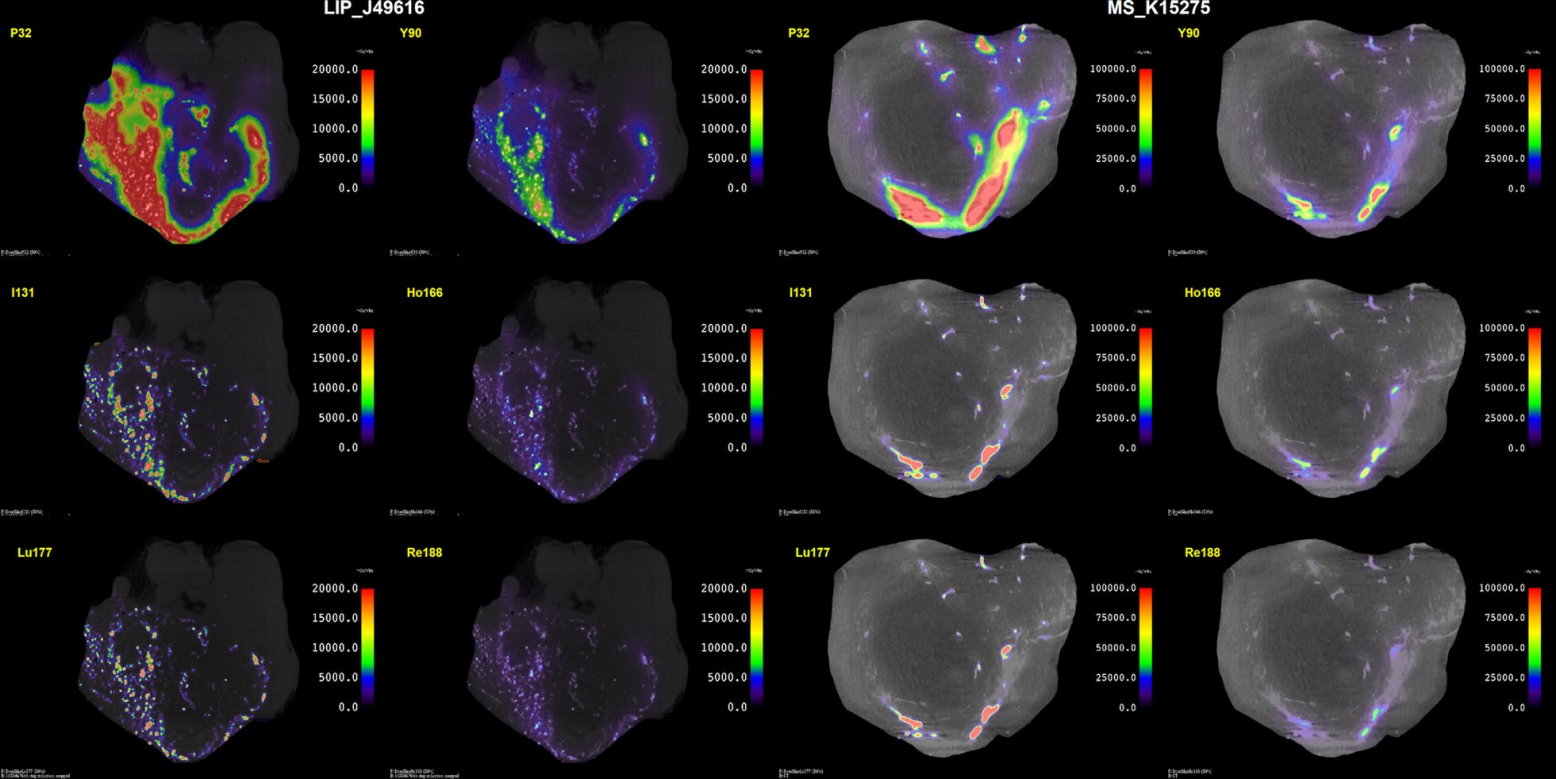
Absorbed dose maps per unit of activity administered to the tumor (*S*/*λ*) in mGy∙MBq^−1^ for ^32^P, ^90^Y, ^131^I, ^166^Ho, ^177^Lu, and ^188^Re in tumors LIP_J49616 (left) and MS_K15275 (right)

**Fig. 5:**
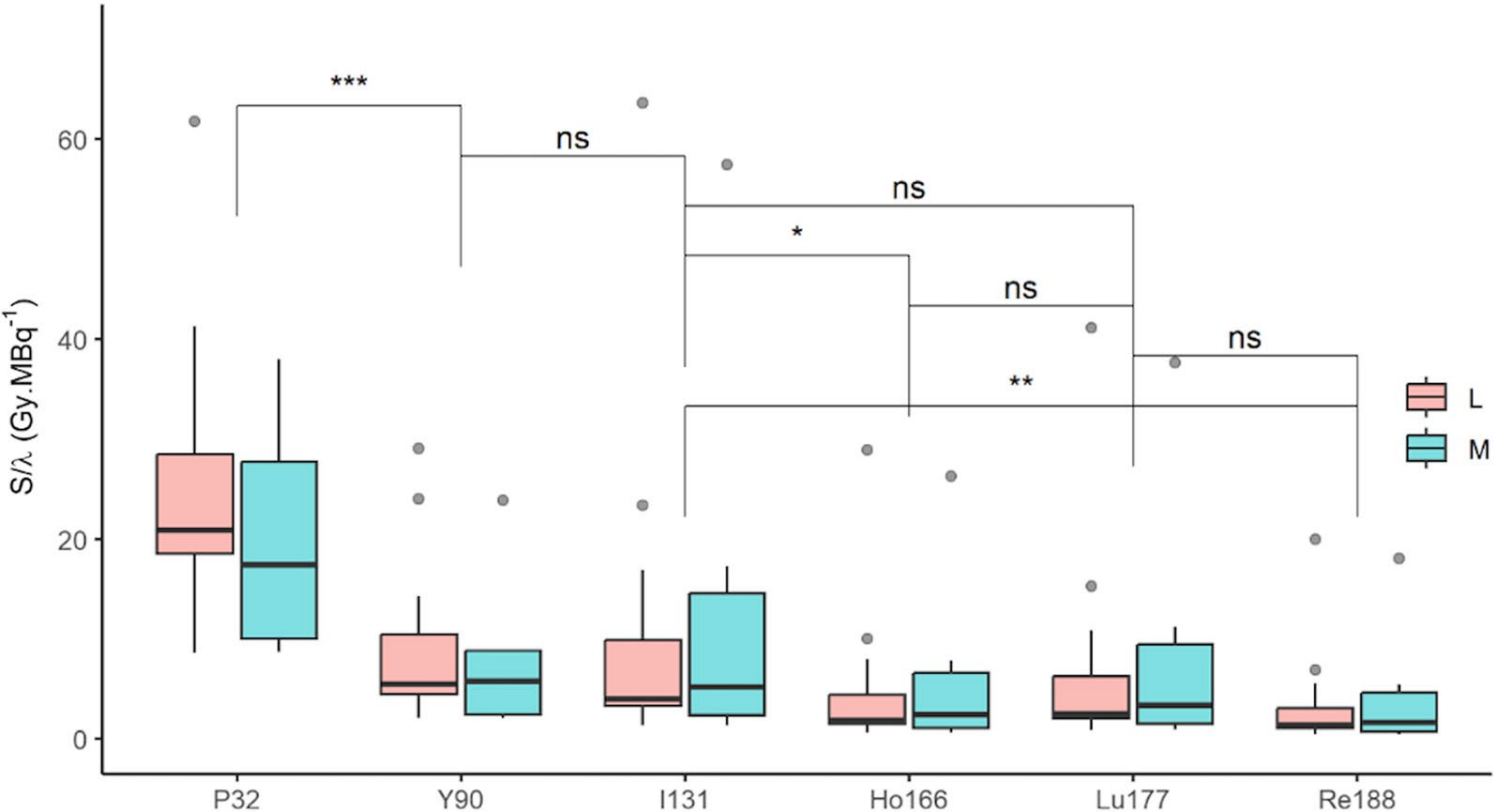
* S*/*λ* in Gy.MBq^−1^ results presented as boxplots for the L and M groups, for ^32^P, ^90^Y, ^131^I, ^166^Ho, ^177^Lu and ^188^Re. *Indicates the difference is significant, while ns indicates it is non-significant

Figure 4 illustrates the absorbed dose maps calculated for tumors of L group and M group. The average *S*/*λ* values are 30% to 40% greater for the M group compared to the L group, but without significance: the greater hypothesis with the Wilcoxon test resulted in *p* values from 0.82 to 1.0. The results are presented as boxplots in Fig. 5, where one can see clearly the significantly greater *S*/*λ* values obtained for ^32^P compared to other radionuclides in the L and M groups. As an example, for ^32^P, *S*/*λ*  = 63 ± 94 Gy∙MBq^−1^ and 86 ± 127 Gy∙MBq^−1^ and for ^90^Y, *S*/*λ*  = 15 ± 22 Gy∙MBq^−1^ and 20 ± 29 Gy.MBq^−1^ in the L and M groups, respectively. Interestingly, the *S*/*λ* values of ^90^Y are not statistically different from those of ^131^I, despite the difference in Δ_β_/λ as reported in Table 2. No statistical difference was found between ^166^Ho, ^177^Lu, and ^188^Re. All *S*/*λ* data are available as Additional file 1.

**Fig. 6:**
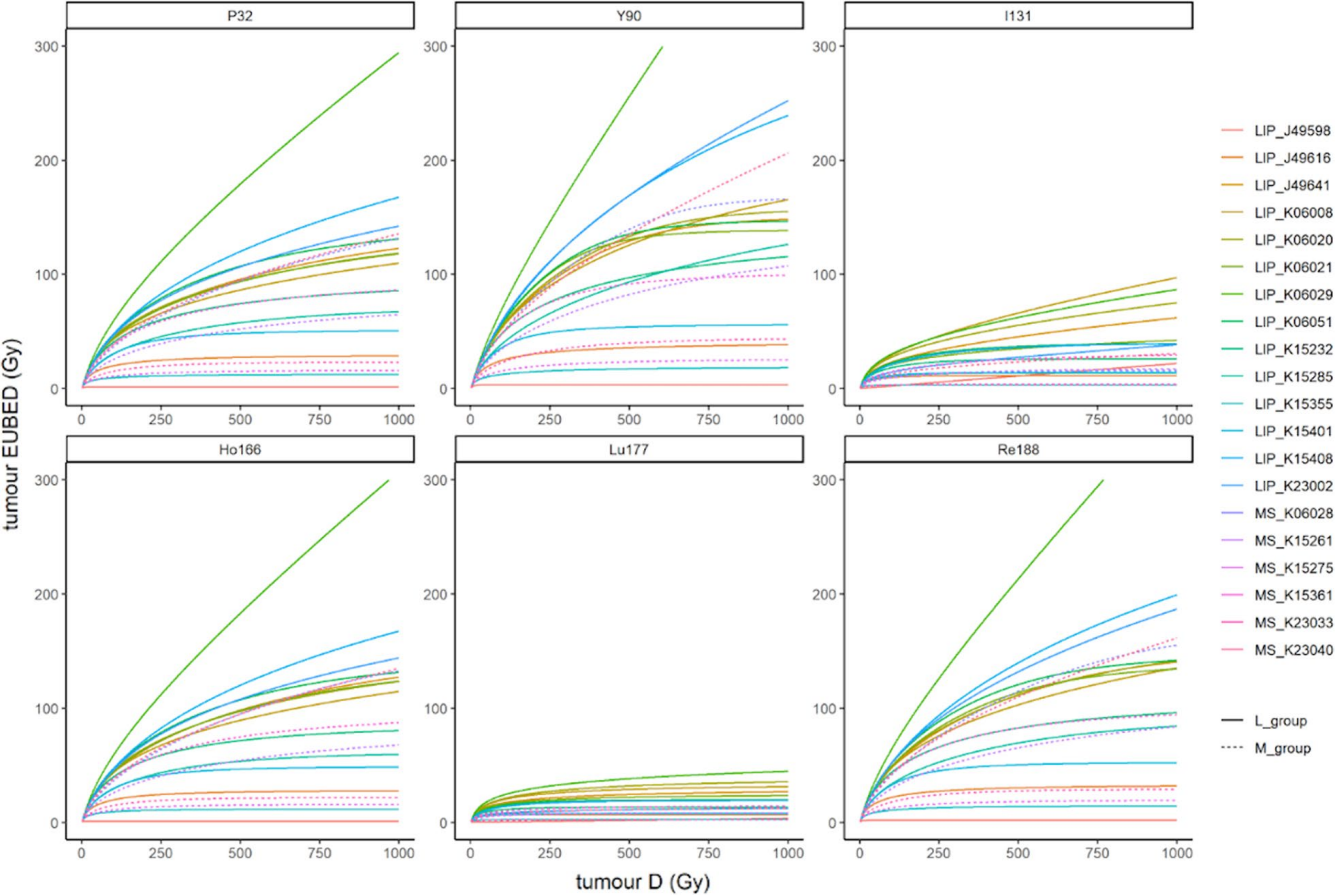
Tumor EUBED in Gy as a function of tumor-absorbed dose *D* in Gy for ^32^P, ^90^Y, ^131^I, ^166^Ho, ^177^Lu, and ^188^Re

Figure 6 shows the variation of EUBED as a function of the absorbed dose (D). One can see that the EUBED values tend to be much lower for ^131^I and ^177^Lu compared to other radionuclides. For some tumors, the relationship between D and EUBED becomes linear over a value of D that depends on the radionuclide. It can be noted that 3 of 14 tumors of the L group curves are above the ones belonging to M group, which indicates a sign for the EUBED of the L group to be greater than that of the M group.

**Fig. 7:**
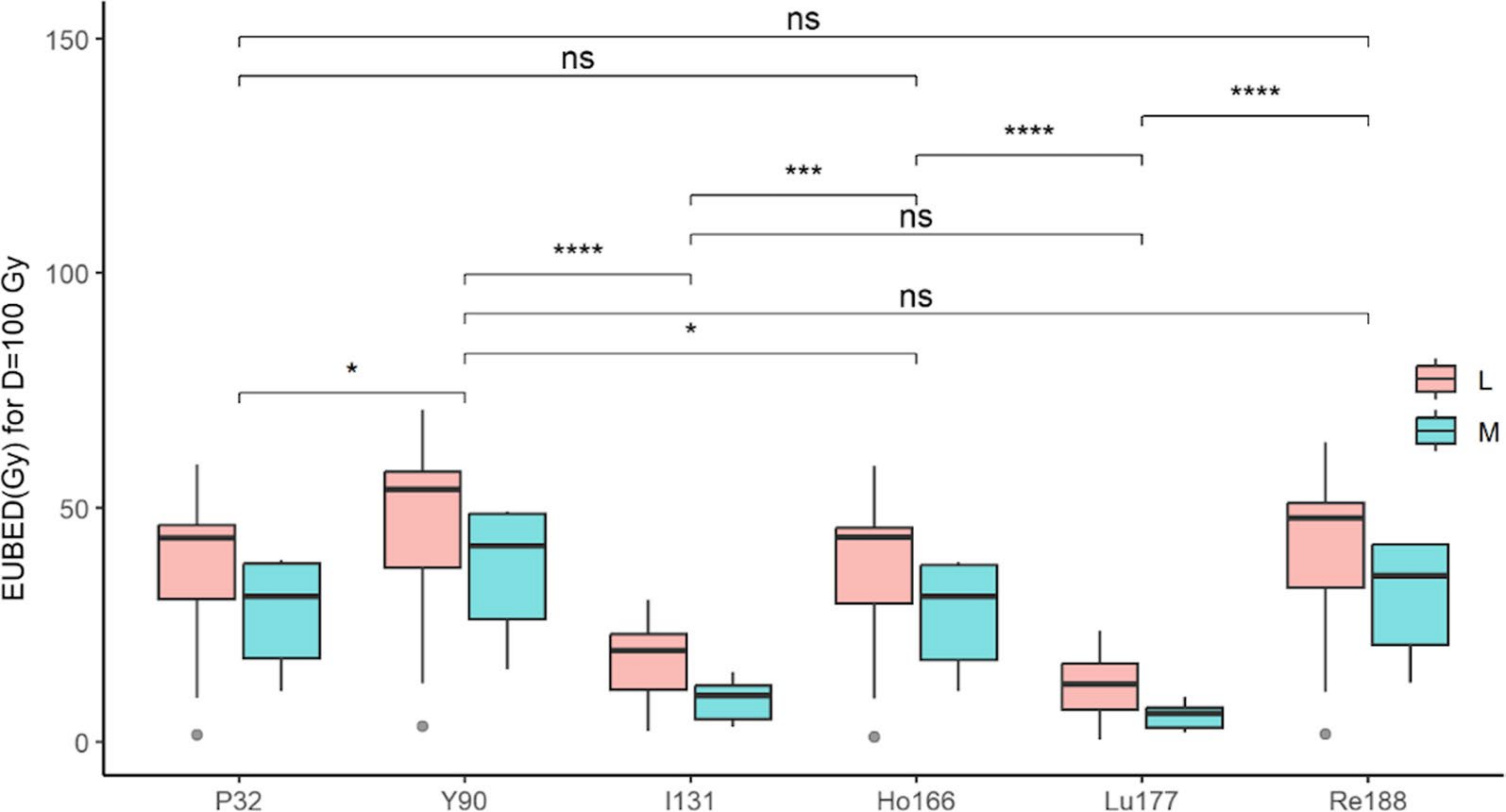
EUBED in Gy for D = 100 Gy values presented as boxplots for the L and M groups, for ^32^P, ^90^Y, ^131^I, ^166^Ho, ^177^Lu, and ^188^Re. * indicates the difference is significant, while ns indicates it is non-significant

Indeed, average EUBED values for D = 100 Gy were higher for the L group (no statistical significance) except for ^177^Lu (*p* = 0.03), see Table 3. The highest average EUBED was obtained for ^90^Y with 45 ± 20 Gy in the L group and 37 ± 15 Gy in the M group. The lowest values were obtained for ^131^I and ^177^Lu with, respectively, 18 ± 9 Gy and 12 ± 7 in the L group and 9.0 ± 4.8 Gy and 5.6 ± 3.0 in the M group. In between, the EUBED values of ^32^P, ^166^Ho, ^188^Re were not statistically different with, respectively, 37 ± 16 Gy, 36 ± 16 Gy and 40 ± 18 Gy in the L group and 28 ± 12 Gy, 27 ± 12 Gy and 31 ± 13 Gy in the M group. The comparative results between radionuclides are presented as boxplots in Fig. 7.

**Table 3:** EUBED in Gy for *D* = 100 Gy values for the L and M groups and following radionuclides: 32P, 90Y, 131I, 166Ho, 177Lu, 188Re

	EUBED (Gy) for *D* = 100 Gy					
	^32^P	^90^Y	^131^I	^166^Ho	^177^Lu	^188^Re
LIP_J49598	1.47	3.42	2.30	0.99	0.39	1.74
LIP_J49616	20.0	24.9	9.81	19.7	6.48	21.8
LIP_J49641	43.8	53.7	23.2	43.6	16.9	47.7
LIP_K06008	43.4	54.1	29.2	43.8	19.6	47.8
LIP_K06020	45.6	55.2	27.5	45.4	20.6	49.2
LIP_K06021	44.0	56.9	20.8	44.0	16.1	48.9
LIP_K06029	59.3	70.9	30.3	58.9	23.7	63.9
LIP_K06051	47.9	57.9	22.6	47.2	14.8	51.6
LIP_K15232	40.7	48.9	18.3	39.9	11.1	43.7
LIP_K15285	30.4	39.6	10.9	29.4	7.05	33.7
LIP_K15355	9.45	12.5	3.07	9.27	2.37	10.6
LIP_K15401	30.7	36.3	11.4	29.8	6.83	32.7
LIP_K15408	47.8	59.1	21.1	47.1	13.6	51.9
LIP_K23002	46.5	60.3	14.3	45.8	8.13	51.7
Mean ± sd	36.5 ± 16	45.3 ± 20	17.5 ± 9	36.1 ± 16	12.0 ± 7	39.8 ± 18
MS_K06028	36.6	49.2	10.3	36.3	6.43	41.4
MS_K15261	25.6	35.3	9.43	26.0	5.90	29.5
MS_K15275	10.8	15.6	3.20	10.9	1.99	12.6
MS_K15361	38.6	48.4	15.0	38.4	9.65	42.4
MS_K23033	15.2	23.0	3.27	14.8	1.95	17.8
MS_K23040	38.8	48.8	12.5	38.2	7.67	42.3
Mean ± sd	27.6 ± 12	36.7 ± 15	8.96 ± 4.8	27.4 ± 12	5.60 ± 3.0	31.0 ± 13
Wilcoxon *p*	0.11	0.13	0.08	0.11	0.04	0.11

Wilcoxon *p* values were calculated for each radionuclide between the L and M groups, with the hypothesis of *L* values greater than *M*

## Discussion

Radioembolization of liver tumors is a well-established treatment strategy for HCC [2]. Currently, there are 3 major options that are based on microspheres [30]: ^90^Y-resin-microspheres, ^90^Y-glass-microspheres, and ^166^Ho-PLGA-microspheres. As reported by Bouvry et al. [15], other compounds based on Lipiodol® ultra-fluid were developed and evaluated, but to date no direct comparison is available in terms of biodistribution and dosimetry.

This study aimed at comparing the biodistributions of Lipiodol® and microspheres in a VX2 tumor model implanted in rabbits. The biodistributions were assessed though histology and µCT, and absorbed dose distributions were simulated for radionuclides of interest in radioembolization, i.e., ^32^P, ^90^Y, ^131^I, ^166^Ho, ^177^Lu, and ^188^Re. The distributions were analyzed visually and using first-order radiomics, while the absorbed dose distributions were completed by radiobiological modeling to compare biological effective dose (BED).

The analysis of the µCT images showed that the Lipiodol® ultra-fluid perfused the large and small vessels, feeding the tumor parenchyma but also diffuses in the extravascular compartment, while the microspheres stay strictly intravascular. This observation of Lipiodol® ultra-fluid being a more penetrative agent (confirmed by the histology) was consistent with the radiomic analysis showing a significantly greater entropy in the Lipiodol® ultra-fluid group (4.06, *n* = 14) compared to the microspheres (2.67, *n* = 6).

The dosimetry analysis showed that the absorbed dose per activity administered to the tumor (*S*/*λ*) was higher for the M group than for the L group, but without statistical significance. The highest average values were found for ^32^P with 86.3 Gy∙MBq^−1^ in M group and 62.8 Gy∙MBq^−1^ in L group, which was significantly higher than ^90^Y with 19.9 Gy∙MBq^−1^ and 14.9 Gy∙MBq^−1^, respectively. All other radionuclide *S*/*λ* values were below that of ^90^Y. The lowest values were found for ^188^Re with 4.67 Gy∙MBq^−1^ and 3.43 Gy∙MBq^−1^ for M and L groups, respectively.

In order to simulate the biological efficacy of radionuclides, we calculated the equivalent uniform biological effective dose (EUBED), using radiobiological parameters found in the literature. The values of *α* and *β* were issued from clinical data [26]. We found that the mean EUBED values for a tumor-absorbed dose of 100 Gy were systematically higher for the L group than for the M group. This suggests that the more distal penetration of Lipiodol® ultra-fluid should have an impact on tumor treatment efficacy, which may be expected superior to that of microspheres. Regarding the comparison between radionuclides, EUBED values were significantly higher for ^90^Y than all other radionuclides but ^188^Re. The lowest EUBED values were found for ^131^I and ^177^Lu.

Aside from these differences, the Lipiodol® ultra-fluid does not remain in the healthy liver parenchyma [13, 31, 32] contrary to microspheres that are blocked by microvessels, regardless of being tumoral or healthy tissue feeders. This could be an advantage for Lipiodol® ultra-fluid as a radionuclide carrier for radioembolization treatments where more than a single segment of the liver needs to be treated. Different retention mechanisms are currently evoked in the tumor (accumulation of the product in the peri-tumoral sinuses by an embolization mechanism [27], modification of the membrane potential or of the permeability of the tumor vessels [31], slower elimination linked to a deficiency in Küpffer cells and lymphatic vessels in the tumor, membrane and then intra-cellular fixation, pinocytosis of Lipiodol® ultra-fluid droplets in HepG2 cells). While the microspheres remain blocked in the microvessels with heterogeneity in targeting the tumor, the slow infusion of radiolabeled Lipiodol® ultra-fluid in the tumor may offer potential for better biological effectiveness while preserving the healthy liver tissues.

This study clearly shows that there are some trends toward a better penetration of Lipiodol® ultra-fluid that may translate into a better radiation efficacy. The comparison of various radionuclides on such a dataset had never been done before. One interesting result is that at an absorbed dose of 100 Gy, the greatest simulated biological efficacy was obtained with ^90^Y and ^188^Re, while the lowest was obtained for ^131^I and ^177^Lu. This can be explained by the longest beta radiation range of ^90^Y and ^188^Re, but also their shortest half-life resulting in a higher dose-rate for a given absorbed dose delivered. Indeed, at higher dose-rate, the cell-killing effect is higher due to lack of reparation capabilities. In between, we found ^32^P, ^166^Ho, whose EUBED values are not statistically different but remain one-third below those of ^90^Y.

Our study has several limitations. First, the number of animals differ between groups and the imaging points are not equal in each group. This is due to the primary endpoint, which was to study the biodistribution kinetic of Lipiodol® ultra-fluid in VX2 tumors, which limits the interpretation of these results. Another limitation is the choice of model since there is no HCC model in rabbits. Nevertheless, although not of hepatic origin, the VX2 model is commonly used as an alternative for interventional radiotherapy studies [33].

## Conclusion

The aim of this study was to compare the ability of Lipiodol® ultra-fluid and microspheres to target the tumor tissues for radioembolization purposes. The images obtained from µCT on ex vivo tumors have demonstrated the ability of Lipiodol® ultra-fluid to penetrate the tumor more extensively than the microspheres and confirm that Lipiodol® ultra-fluid remains in the tumor compartment for at least 12 days. This ability translated into a higher simulated EUBED than for microspheres, hence the potential of Lipiodol® ultra-fluid for a better efficacy. This study allowed also to confirm that ^90^Y might be the best candidate radionuclide for radioembolization, either with Lipiodol® ultra-fluid or microspheres, in terms of efficacy, but ^32^P, ^166^Ho, and ^188^Re can achieve close results, contrary to ^131^I and ^177^Lu. The results of this study could be used to investigate the development of novel radioembolization agents with Lipiodol® ultra-fluid as a radioactivity delivery agent and to help transposing the clinical results from an agent to another.

### Supplementary Information

**Additional file 1. **Supplemental data: boxplots and correlation maps of the radiomic analysis, and absorbed dose estimations tabulated and represented as dose-volume histograms.

## Data Availability

All data generated or analyzed during this study are included in this published article and its supplementary files.

## Abbreviations

3D: Tri-dimensional
BCLC: Barcelona Clinic Liver Cancer
BED: Biological effective dose
CSDA: Continuous slowing-down approximation
DPK: Dose-point kernel
EUD: Equivalent uniform dose
EUBED: Equivalent uniform biological effective dose
HCC: Hepatocellular carcinoma
MIRD: Medical internal radiation dose
µCT: Micro-computerized tomography
SSS: Super-six sulfur
