# Supplementary material for: Biological efficacy of simulated radiolabeled Lipiodol® ultra-fluid and microspheres for various beta emitters: study based on VX2 tumors
Source: EJNMMI Res. 2023 Nov 23;13:101. doi: 10.1186/s13550-023-01051-9 (PMC10667182; doi:10.1186/s13550-023-01051-9)
Supplement: Supplementary file 1 — Additional file 1. Supplemental data: boxplots and correlation maps of the radiomic analysis, and absorbed dose estimations tabulated and represented as dose-volume histograms. [file 13550_2023_1051_MOESM1_ESM.pdf]

Supplemental figure 1 : Radiomics boxplots for C group (n=3, no injection), L group (n=14, Lipiodol® ultra-fluid), M group (n=6, microspheres). Radiomics features calculated in the tumor with SlicerRadiomics extension of 3DSlicer.

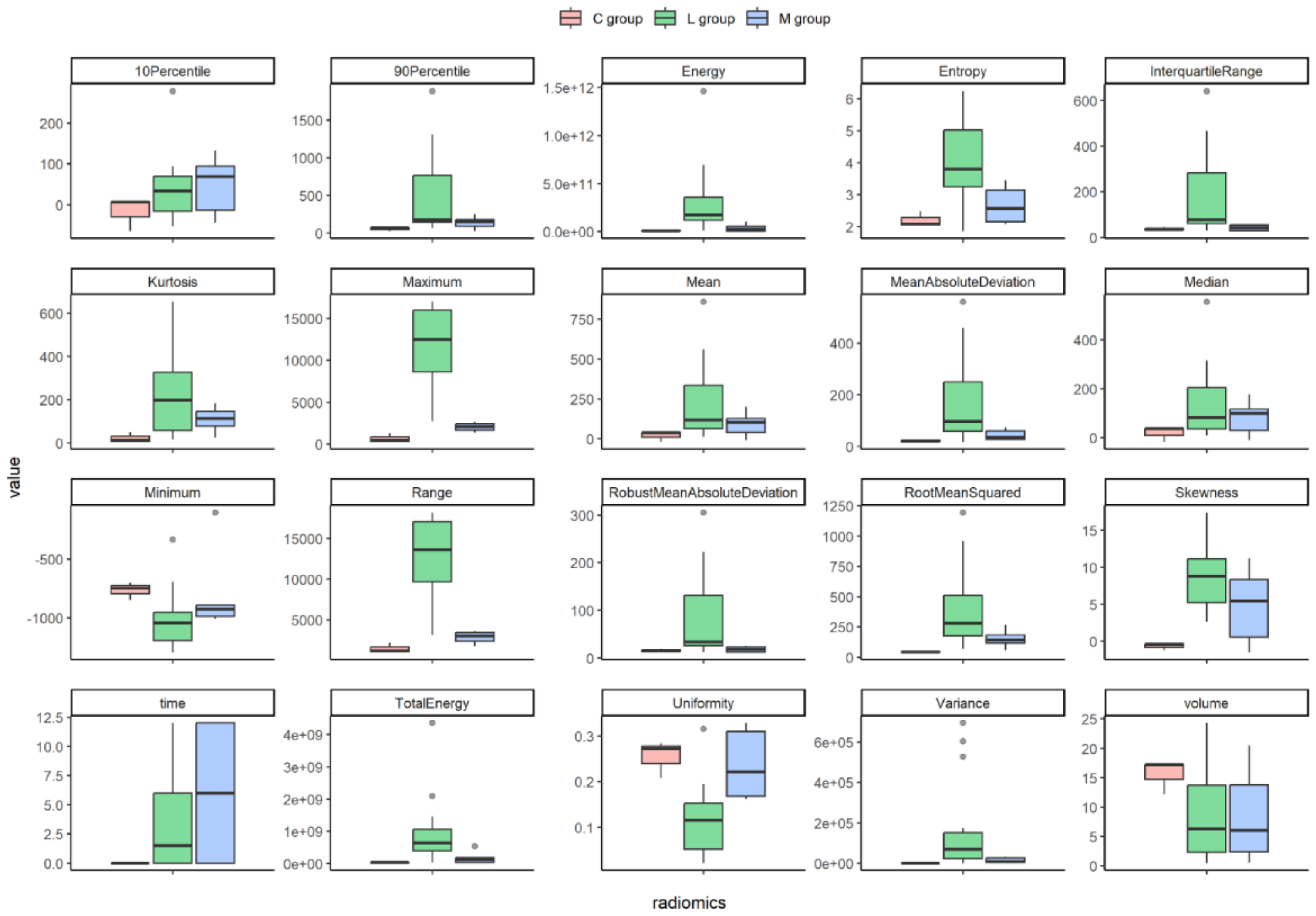

Supplemental figure 2 : Correlation map for L group (n=14, Lipiodol® ultra-fluid). Radiomics features calculated in the tumor with SlicerRadiomics extension of 3DSlicer.

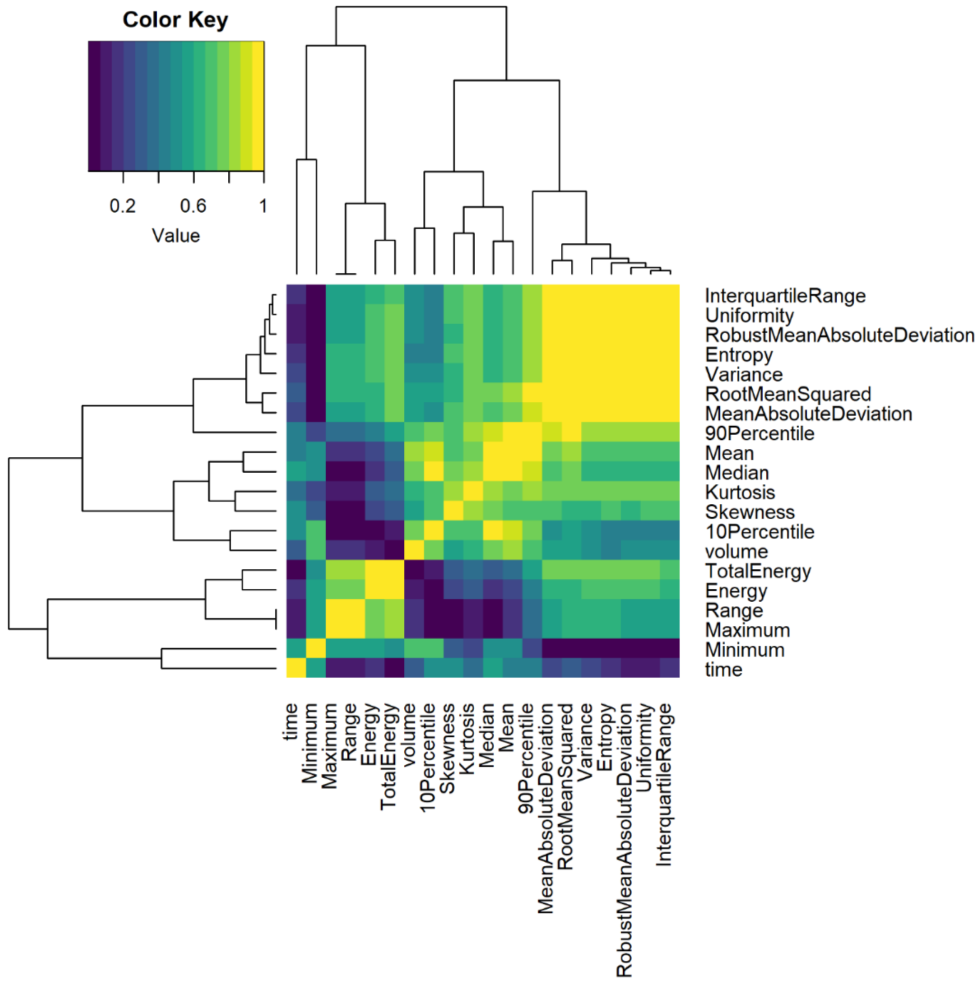

Supplemental figure 3 : Correlation map for M group (n=6, microspheres). Radiomics features calculated in the tumor with SlicerRadiomics extension of 3DSlicer.

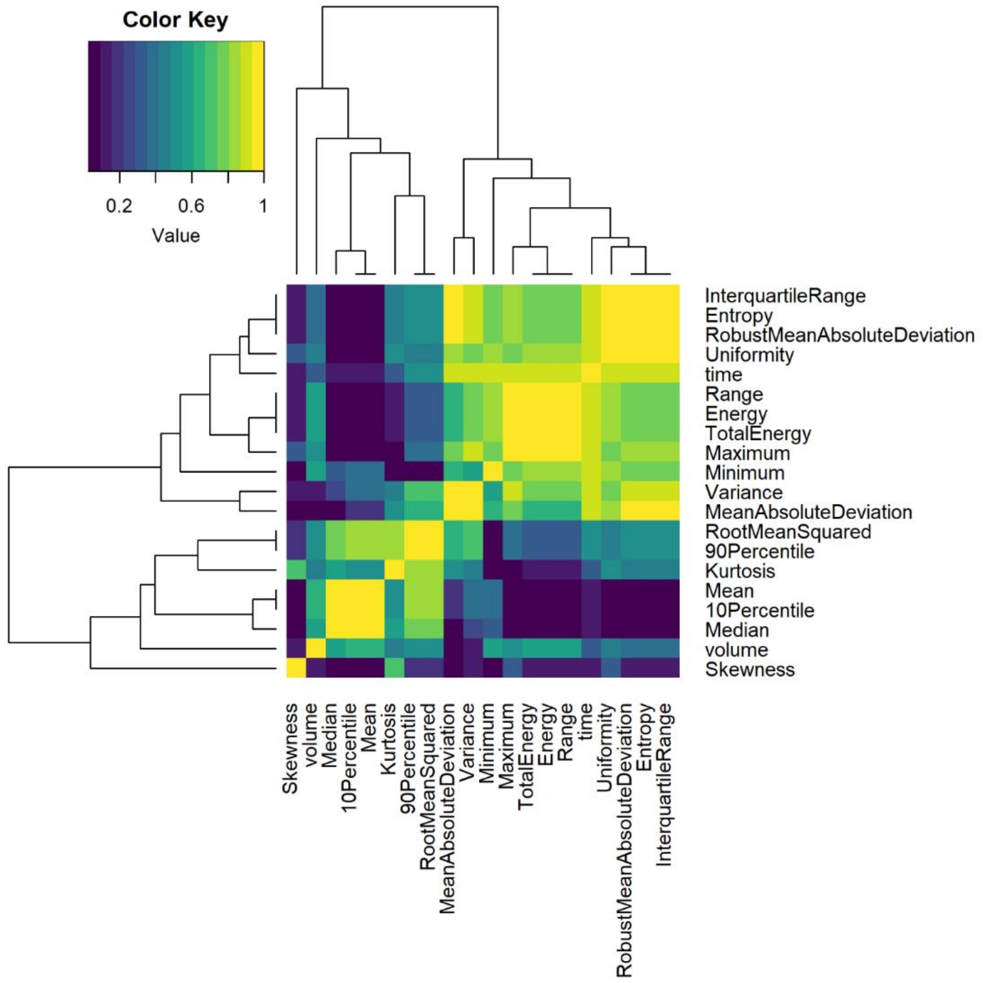

Supplemental figure 4: Dose volume histograms in Gy/MBq for all tumors explanted.

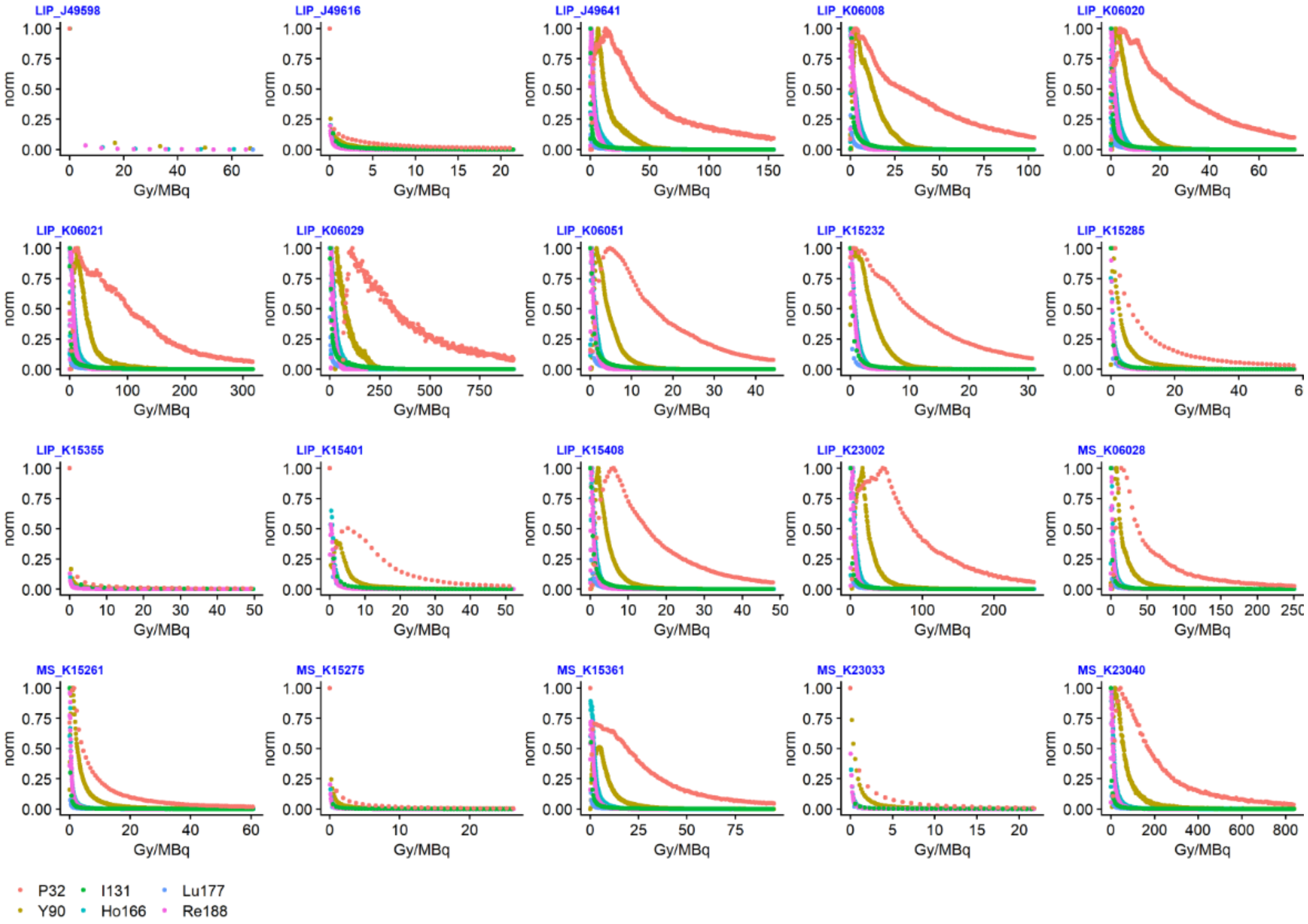

Supplemental table 1: S/λ values for the L and M groups and following radionuclides: ^{32}P, ^{90}Y, ^{131}I, ^{166}Ho, ^{177}Lu, ^{188}Re. Wilcoxon p-values were calculated for each radionuclide between the L and M groups, with the null hypothesis.

	S/λ (Gy.MBq⁻¹)					
	**^{32}P**	**^{90}Y**	**^{131}I**	**^{166}Ho**	**^{177}Lu**	**^{188}Re**
LIP_J49598	27.2	8.38	3.93	1.96	2.63	1.55
LIP_J49616	8.60	2.12	1.36	0.670	0.85	0.476
LIP_J49641	61.8	14.3	11.1	4.86	7.10	3.35
LIP_K06008	41.3	10.5	6.34	3.24	3.81	2.32
LIP_K06020	29.7	6.88	5.18	2.33	3.29	1.61
LIP_K06021	127	29.1	23.4	10.0	15.27	6.88
LIP_K06029	368	85.6	63.7	28.9	41.15	20.0
LIP_K06051	17.8	4.20	2.96	1.39	1.83	0.97
LIP_K15232	12.4	3.02	1.95	0.963	1.19	0.68
LIP_K15285	22.9	5.51	4.02	1.78	2.44	1.26
LIP_K15355	19.9	5.08	3.29	1.56	2.07	1.12
LIP_K15401	20.9	5.12	3.48	1.62	2.06	1.15
LIP_K15408	19.3	4.52	3.30	1.51	2.09	1.05
LIP_K23002	102	24.0	16.9	7.98	10.87	5.56
Mean ± sd	62.8 ± 94	14.9 ± 22	10.8 ± 16	4.92 ± 7.4	6.9 ± 11	3.43 ± 5.1
MS_K06028	100	23.9	17.3	7.87	11.3	5.49
MS_K15261	24.3	5.75	3.99	1.90	2.58	1.33
MS_K15275	10.5	2.43	1.83	0.823	1.20	0.57
MS_K15361	38.0	8.81	6.48	2.98	4.15	2.06
MS_K23033	8.70	2.10	1.41	0.676	0.922	0.477
MS_K23040	336	76.2	57.5	26.3	37.6	18.1
Mean ± sd	86.3 ± 127	19.9 ± 29	14.7 ± 22	6.75 ± 9.9	9.6 ± 14	4.67 ± 6.8
Wilcoxon p	1.0	0.97	0.90	1.0	0.84	1.0
